# Anatomic variations of the renal arteries, as characterized by computed tomography angiography: rule or exception? Its usefulness in surgical plannning

**DOI:** 10.1590/0100-3984.2016.49.4e2

**Published:** 2016

**Authors:** David C. Shigueoka

**Affiliations:** 1 Adjunct Professor in the Department of Diagnostic Imaging at the Escola Paulista de Medicina da Universidade Federal de São Paulo (EPM-Unifesp), São Paulo, SP, Brazil. E-mail: sdavid@uol.com.br

In his *Traité d'Anatomie Humaine* , the classical anatomist Testut noted that "the kidneys, as well as other organs that perform important functions, have an extremely rich and complex vasculature"^(1)^. In the previous issue of **Radiologia Brasileira**, Mello Júnior et al.^(2)^ described the normal appearance of the renal arteries and their most common anatomical variations, as observed with computed tomography (CT) angiography. The authors also discussed the technical aspects of implementing CT angiography, the post-processing of images, and the terminology involved, as well as the clinical and surgical implications.

In a recent study employing CT angiography, Çinar et al.^(3)^ identified polar renal arteries in 31.3% of the cases and early bifurcation of the hilar artery in 6.5%, which indicates that such anatomic variants are not merely exceptions, but rather are quite common. Therefore, knowledge of these variants is crucial to surgical planning in the treatment of various pathological conditions.

In the current state of the art, CT angiography is superior to magnetic resonance angiography in the evaluation of the renal vessels, as well as other branches of the aorta^(4)^, particularly in the identification of vessels with a diameter of less than 2 mm^(5)^, although it may be used in the evaluation of renal arteries with acceptable results^(6)^.

Prior to kidney transplantation, the evaluation of a living donor who is a candidate for laparoscopic nephrectomy is one of the main indications for the preoperative study of the renal arteries. The left kidney is most often used because of the technical facility and its longer vascular pedicle, the left renal vein therefore being longer than the right^(7)^. In rare cases, anatomic variations of the renal arteries are an absolute contraindication to performing the kidney transplantation, more than three aberrant arteries being considered a limiting factor. Early bifurcation of the renal artery (i.e., the emergence of segmental branches 1.5-2.0 cm from its origin), which was observed in 13% of the patients evaluated in a study conducted by Munnusamy et al., limits the vascular anastomosis in the recipient^(8)^, and, if necessary, a superior polar artery with a diameter of less than 2 mm can be ligated without significant graft ischemia^(9)^.

Another important indication for the preoperative study of the renal arteries is the evaluation of candidates for the endovascular treatment of aortic aneurysms, using fenestrated or branched stent grafts. In addition to the location and caliber of the hilar arteries, the presence of polar arteries must be known in order to predict possible kidney injury, although the sacrifice of a small-caliber polar artery that could be obliterated by the prosthesis can be acceptable, provided that the ischemic segment kidney is minimal^(10)^.

Mello Júnior et al.^(2)^ also underscored the importance of characterizing any accessory inferior polar artery in the surgical treatment of stenosis at the ureteropelvic junction (UPJ). Although the polar artery is not always the cause of obstruction, its identification is useful in surgical planning, particularly when the procedure will be endoscopic, in which the long longitudinal incision made over the UPJ can injure any vessel in its path, laparoscopic pyeloplasty being an alternative when a polar artery is identified^(11)^. Because UPJ stenosis is typically diagnosed in childhood, magnetic resonance imaging has been used to allow the concurrent assessment of the renal collecting system and of the vascular anatomy, avoiding the disadvantages of using ionizing radiation in this age group, as well as any potential kidney damage caused by the use of iodinated contrast media^(12)^.

The terminology used in the description of the anatomical variations is one of the controversial issues in the literature, due to the diversity of terms used. In the literature, anatomic variations of the renal arteries are described variously as extra, additional, supernumerary, aberrant, anomalous, or incidental renal arteries, as well as (superior and inferior) polar arteries arising from the aorta or simply polar arteries. The *nomina anatomica* in Portuguese is silent on the topic of these structures. Mello Júnior et al.^(2)^ recommended the Portuguese-language terminology proposed by Sampaio et al.^(13)^ and employed by Palmieri et al.^(14)^, both of whom conducted studies in Brazil and who used (the equivalents of) the denominations hilar artery, (superior and inferior) extra-hilar artery, (superior and inferior) polar artery, and early bifurcation, as well as providing a detailed description of each. It should be borne in mind that the Portuguese-language equivalent of the term accessory polar artery (*artéria acessória polar*) is also widely used in Brazil. A consensus on the anatomical terminology employed across the different specialties involved would certainly be well received.

Finally, Mello Júnior et al.^(2)^ provided practical recommendations for the interpretation of CT angiography of the renal arteries, guiding the detailed description, plus measures, which promotes greater consistency in communicating the findings to the surgeon, as previously suggested by other authors^(15)^.

In conclusion, the radiologist plays an important role in the diagnostic and preoperative evaluation of the renal vasculature, contributing to the reduction of complications, as well as promoting the success, of therapeutic interventions.
